# Does treatment with dutasteride or finasteride has impact on renal morphology? Experimental study

**DOI:** 10.1590/ACB360703

**Published:** 2021-09-15

**Authors:** Marcello Henrique Araújo da Silva, João Henrique Duque Estrada, Bianca Martins Gregório, Francisco José Barcellos Sampaio, Diogo Benchimol de Souza

**Affiliations:** 1 MSc. Urogenital Research Unit – Universidade do Estado do Rio de Janeiro - Rio de Janeiro (RJ), Brazil.; 2 MD. Urogenital Research Unit – Universidade do Estado do Rio de Janeiro (UERJ) - Rio de Janeiro (RJ), Brazil.; 3 PhD. Urogenital Research Unit – Universidade do Estado do Rio de Janeiro (UERJ) - Rio de Janeiro (RJ), Brazil.; 4 PhD. Urogenital Research Unit – Universidade do Estado do Rio de Janeiro (UERJ) - Rio de Janeiro (RJ), Brazil.; 5 PhD. Urogenital Research Unit – Universidade do Estado do Rio de Janeiro (UERJ) - Rio de Janeiro (RJ), Brazil.

**Keywords:** Prostatic Hyperplasia, Dutasteride, Finasteride, Kidney, Models, Animal

## Abstract

**Purpose:**

To investigate whether renal modifications occur following treatment with dutasteride or finasteride.

**Methods:**

Twenty-four male rats were divided into three groups: control (that received distilled water), dutasteride (0.5 mg/kg/day), and finasteride (5 mg/kg/day) groups. All administrations were given by gavage for 40 consecutive days. After inducing euthanasia, blood was collected for urea and creatinine analyses, and both the kidneys were collected for stereological analyses of kidney morphology.

**Results:**

Serum urea and creatinine levels were increased in both the finasteride and the dutasteride groups compared with those in the control group. In addition, kidney weight, kidney volume, cortical volume, glomerular volumetric density, and mean glomerular volume were reduced in both treatment groups. Finally, the number of glomeruli per kidney was reduced by 26.8% in the finasteride group and by 51.6% in the dutasteride group compared with that in the control group.

**Conclusions:**

The 5-ARIs finasteride and dutasteride promoted morphological and functional damages in rat kidneys. In addition, rats in the dutasteride group showed more severe renal modifications than those in the finasteride group.

## Introduction

Benign prostatic hyperplasia (BPH) is a disease characterized by the enlargement of prostatic epithelial and stromal tissues and reduced urinary flow, resulting in manifestations commonly known as lower urinary tract symptoms (LUTS)^1^. It is well known that ageing is correlated with BPH, which affects 50% of men older than 50 years old and 90% of men in their 80s^1^^,^ 2.

The first-line pharmacological treatment for BPH indicated by the European Association of Urology and the American Urological Association comprises 5-alpha reductase inhibitors (5-ARIs)^3^^,^ 4. This class of drugs prevents the conversion of testosterone to dihydrotestosterone (DHT), which is the most active androgen^2^^,^ 5. As the prostate is an androgen-dependent organ, the reduction of DHT levels is often enough to reduce prostate volume and treat clinical symptoms associated with BPH.

However, treatment with 5-ARIs may result in adverse effects. For example, histomorphometrical alterations of the corpus cavernosum associated with erectile function have been previously described^5^^-^ 7. Furthermore, 5-ARIs decrease the expression of endothelial growth factor (VEGF) and inhibit angiogenesis in the prostate, which explains decreased bleeding observed following prostatectomies^8^^,^ 9.

Nevertheless, information regarding renal damage associated with 5-ARIs in the literature is limited. Recently, it has been shown that finasteride may cause renal damage, inducing apoptosis and tubular changes^10^. It was further observed that this drug reduces VEGF and vascularization of renal tissue of diabetic rats^11^. However, it is not known whether treatment with 5-ARIs can modify glomerular morphology.

Thus, the objective of this study was to investigate whether treatment with the 5-ARIs dutasteride or finasteride can promote renal (specifically, glomerular) morphological modifications.

## Methods

This project was approved by the local ethics committee under the protocol number CEUA-041/2017.

Twenty-four male Wistar Kyoto rats were used in this study. All animals were bred in the Urogenital Research Unit facility and were housed in a room with controlled temperature (23°C ± 1°C), artificial 12-hour dark-light cycle (lights on from 7 a.m. to 7 p.m.), and free access to the standard rat chow and water.

Rats (4 months of age) were randomly assigned to one of three groups:

- Ctrl (n = 8): the control one, received distilled water;
- Dut (n = 8): received 0.5 mg/kg/day of dutasteride (Dastene, Aché, Indaiatuba, SP, Brazil)^5^^,^ 6;
- Fin (n = 8): received 5 mg/kg/day of finasteride (Finasterida, Eurofarma, São Paulo, SP, Brazil)^5^^,^ 10 ^,^ 12.

The drugs and the distilled water were administered by gavage for 40 consecutive days.

After the experimental period, animals were euthanized by overdose of sodium thiopental (Thiopentax 1 g, Cristália, Itapira, SP, Brazil). Immediately after death, blood was collected by cardiac puncture and centrifuged, and isolated serum was preserved at -20°C. In addition, both kidneys were collected and fixed in 4% buffered formaldehyde.

Serum creatinine and urea levels were measured with kits (kinetic creatinine and enzyme urea; Bioclin, Belo Horizonte, MG, Brazil).

Kidneys were weighed and their volumes measured by Scherle’s method^13^^,^ 14. Left kidneys, transversely sliced at a thickness of 2 mm into sequential sections, were used for determining the cortical-medullar ratio using Cavalieri’s principle^15^^,^ 16. The absolute cortical volume (CV) was calculated by multiplying the cortical-medullary ratio by the renal volume^13^.

Fragments from the right kidneys were collected and routinely processed for paraffin embedding. Sections of 5-µm thickness were obtained and stained with hematoxylin and eosin. Twenty-five randomly selected histological fields of the cortex of each kidney were analyzed. These fields were photographed using a digital camera (DP70, Olympus, Tokyo, Japan) coupled with a microscope (BX51, Olympus, Tokyo, Japan) under 200× magnification.

Glomerular volumetric density (Vv[glom]), which indicates the proportional volume occupied by the glomeruli in the cortex, was estimated by the point-counting technique with an M42 test system^17^^,^ 18. Volume-weighted glomerular volume (VWGV), which indicates the mean volume of the glomeruli, was estimated by the point-sampled intercepts method by analyzing 50 glomeruli per animal^15^^,^ 17 ^,^ 18. Quantitative analyses of Vv[glom] and VWGV were performed using the ImageJ software (version 1.46r, National Institutes of Health, Bethesda, United States). The total number of glomeruli per kidney was estimated by dividing the product of the cortical volume and Vv[glom] by VWGV^13^^,^ 17 ^,^ 18.

One-way analysis of variance (ANOVA) with Bonferroni’s post-hoc test was used to compare mean values, with the significance level set at p<0.05. All analyses were performed using the GraphPad Prism software (version 5.0, San Diego, CA, United States).

## Results

Urea serum levels increased by 152.5% in the Dut group and 81.7% in the Fin group compared with those in the Ctrl group. Creatinine serum levels were also increased by 166.3% in Dut group and by 124.4% in Fin group compared with those in Ctrl group. All numerical data are presented in Table 1.

**Table 1:** Serological and renal morphological data of rats receiving dutasteride or finasteride vs. control*.

	Ctrl	Dut	Fin	p-value
Serum creatinine (mg/dL)	0.86 ± 0.30	2.29 ± 0.69^a^	1.93 ± 0.80^a^	0.0041
Serum urea (mg/dL)	28.14 ± 10.44	71.05 ± 17.42^a^	51.14 ± 2.42^a^	<0.0001
Kidney weight (g)	1.17 ± 0.09	1.00 ± 0.15^a^	0.90 ± 0.04^a^	<00002
Kidney volume (ml)	1.15 ± 0.09	0.98 ± 0.15^a^	0.87 ± 0.04^a^	<0.0001
Cortical-medullary ratio (%)	84.75 ± 2.05	56.88 ± 9.25^a^	78.00 ± 1.41^a^	<0.0001
Cortical volume (ml)	0.97 ± 0.08	0.57 ± 0.18^a^	0.68 ± 0.03^a^	<0.0001
Vv[Glom] (%)	10.46 ± 0.79	6.62 ± 0.70^a^	7.35 ± 0.68^a^	<0.0001
VWGV (×10^5^µm^3^)	10.35 ± 6.72	8.17 ± 5.37^a^	7.12 ± 2.57^a^	<0.0001
Glomeruli per kidney (×10^4^ µm^3^)	9.54 ± 2.23	4.62 ± 1.56^a^	6.98 ± 5.30^a^	<0.0001

Ctrl: Control group, composed of Wistar Kyoto rats that received distilled water; Dut: dutasteride group, composed of rats that received dutasteride; Fin: finasteride group, composed of rats that received finasteride; Vv[Glom]: glomerular volumetric density; VWGV: volume-weighted glomerular volume; adifferent from Ctrl;

* data presented as mean ± standard deviation.

Kidney weights in the Dut and Fin groups decreased by 14.5 and 23.1%, whereas kidney volumes decreased by 14.8 and 24.3%, respectively, compared with those in the Ctrl group.

Cortical-medullary ratio decreased by 32.9% in the Dut group and by 8% in the Fin group compared with that in the Ctrl group. Regarding the absolute cortical volume, the Dut group had a decrease of 41.2% and the Fin group had a decrease of 29.9% compared with the Ctrl group.

**Figure 1:**
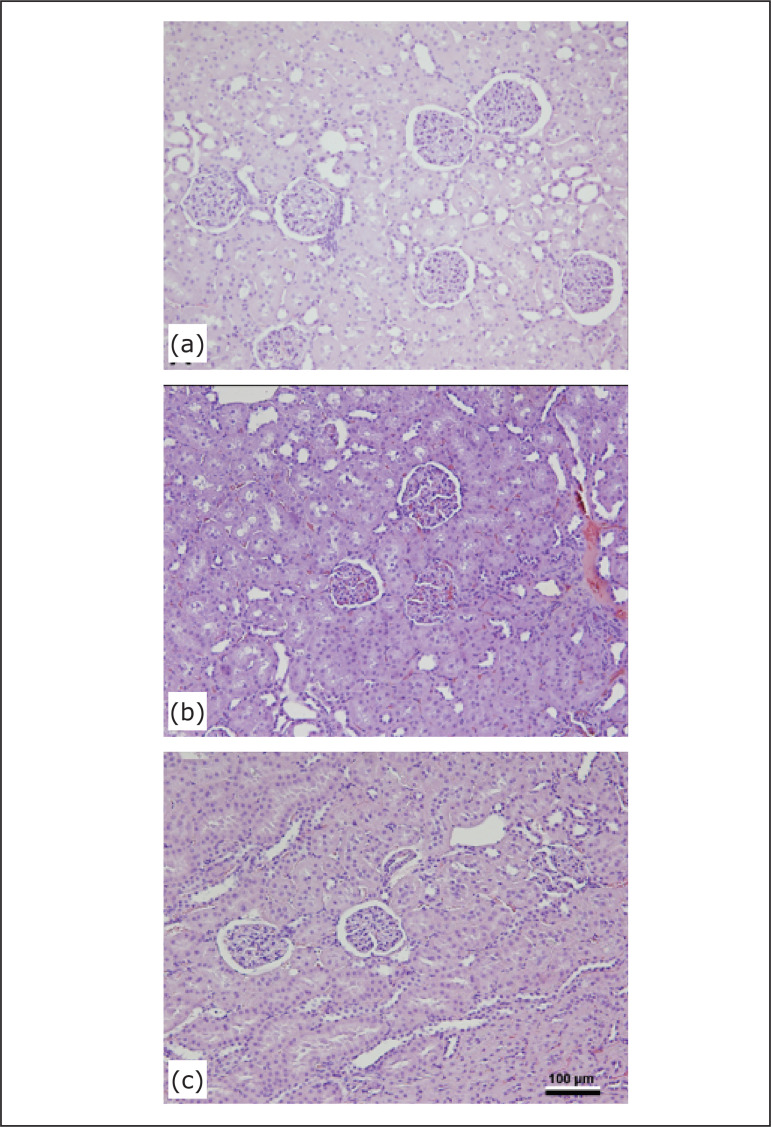
Photomicrographs of renal cortex from the experimental groups: (a) control group, composed of Wistar Kyoto rats that received distilled water; (b) dutasteride group, composed of rats that received dutasteride; (c) finasteride group, composed of rats that received finasteride hematoxylin and eosin, 200x.

In addition, Vv[Glom] and VWGV were reduced in both Dut and Fin groups compared with the ones in the Ctrl group. Animals receiving dutasteride had 36.7% reduction in Vv[Glom] and 21.1% reduction in VWGV. Rats from the Fin group showed 29.7% reduction in Vv[Glom] and 31.2% reduction in VWGV. Figure 1 shows representative images of renal cortex from the groups.

Finally, the number of glomeruli per kidney was reduced by 51.6% in the Dut group and 26.8% in the Fin group compared to the Ctrl group. This represents a loss of approximately 49.200 and 25.600 glomeruli per kidney, caused by the treatment with dutasteride and finasteride, respectively.

## Discussion

The present study is the first to demonstrate that 5-ARIs treatment induces reduction in the number of glomeruli in rats. It is well known that the treatment of BPH or alopecia with 5-ARIs may be associated with side effects. These side effects are mainly seen in the penis and characterized by morphological alterations in the corpora cavernosum and transient or permanent erectile disfunction^5^^-^ 7. However, information regarding side effects in other organs is scarce, especially in tissues not typically recognized as androgen-dependent.

Several conditions can lead to reduction in the number of glomeruli, including stress, renal ischemia, radiofrequency ablation, hypertension, and diabetes^13^^-^ 15 ^,^ 17 ^-^ 20. All these conditions are associated with an increased risk of chronic kidney disease. Moreover, the relationship between decreased glomeruli and decreased glomerular filtration rate is well known^21^. Thus, the quantification of the number of glomeruli, which corresponds to the number of nephrons, becomes a useful and sensitive method to morphologically evaluate possible damage to renal function.

Renal function is not always immediately impaired by glomerular loss. Previous studies have demonstrated considerable loss of glomeruli without changes in serum levels of urea or creatinine^22^^,^ 23. Although the loss of glomeruli is irreversible^19^^,^ 24, unaffected glomeruli can increase their filtration rate, thus keeping the renal biomarkers at a normal level^25^. In the present study, an increase in serum urea and in creatinine levels was observed, indicating that renal damage caused by 5-ARIs was greater than the hyperfiltration capacity of the remaining glomeruli.

Some studies have shown that treatment with 5-ARIs causes decrease in VEGF expression, an increase in collagen in the renal medullar zone, and renal cell apoptosis^9^^-^ 11. DHT is known to regulate several functions in androgen-dependent organs, and the deprivation of this hormone (which is inhibited by 5-ARIs) can promote changes in the urogenital system^2^^,^ 5 ^-^ 7. In addition, it has previously been shown that DHT reduction affects both angiogenesis and VEGF levels in the prostate^26^^-^ 28. Future studies that add insight into the mechanisms underlying DHT and 5-ARI activity in renal tissue are warranted.

In our study, we observed that the number of glomeruli decreased in animals treated either with dutasteride or finasteride, with a greater reduction seen in the animals treated with dutasteride. The fact that this drug acts on more isoforms of the 5-alpha-reductase enzyme^2^^,^ 5 may explain the greater renal damage observed in the present study. Thus, in patients whose renal function is an important aspect to be considered, the use of finasteride may be preferred over dutasteride. The use of alpha-1 blockers may also be a good option in these cases. However, there is evidence that this class of drugs promotes various adverse effects, including renal adverse ones^29^^,^ 30.

Although rodents are widely used, the use of an animal model can be considered a limitation of the present study, as the results cannot be directly extrapolated to humans. Future clinical studies that add knowledge regarding the possible kidney damage caused by 5-ARIs are warranted. Furthermore, studies with longer follow-up periods are required to better understand the long-term renal effects of finasteride and dutasteride.

## Conclusions

The 5-ARIs finasteride and dutasteride promote morphological modifications in the kidneys with biomarker alterations in a rodent model. Dutasteride showed more severe modifications than finasteride. If confirmed in humans, these findings should be considered when choosing BPH treatment, especially in patients with a history of renal disease.
